# Vemurafenib in Langerhans cell histiocytosis: report of a pediatric patient and review of the literature

**DOI:** 10.18632/oncotarget.25277

**Published:** 2018-04-24

**Authors:** Anne Heisig, Jan Sörensen, Stefanie-Yvonne Zimmermann, Stefan Schöning, Dirk Schwabe, Hans-Michael Kvasnicka, Raphaela Schwentner, Caroline Hutter, Thomas Lehrnbecher

**Affiliations:** ^1^ Division of Pediatric Hematology and Oncology, Hospital for Children and Adolescents, Johann Wolfgang Goethe-University, Frankfurt, Germany; ^2^ Division of Stem Cell Transplantation and Immunology, Hospital for Children and Adolescents, Johann Wolfgang Goethe-University, Frankfurt, Germany; ^3^ Institute of Pathology, Johann Wolfgang Goethe-University, Frankfurt, Germany; ^4^ St. Anna Kinderspital, Vienna, Austria

**Keywords:** Langerhans cell histiocytosis, LCH, child, vemurafenib, BRAF

## Abstract

Selective BRAF inhibitors such as vemurafenib have become a treatment option in patients with Langerhans cell Histiocytosis (LCH). To date, only 14 patients receiving vemurafenib for LCH have been reported. Although vemurafenib can stabilize the clinical condition of these patients, it does not seem to cure the patients, and it is unknown, when and how to stop vemurafenib treatment. We present a girl with severe multisystem LCH who responded only to vemurafenib. After 8 months of treatment, vemurafenib was tapered and replaced by prednisone and vinblastine, a strategy which has not been described to date. Despite chemotherapy, early relapse occurred, but remission was achieved by re-institution of vemurafenib. Further investigation needs to address the optimal duration of vemurafenib therapy in LCH and whether and which chemotherapeutic regimen may prevent disease relapse after cessation of vemurafenib.

## INTRODUCTION

Langerhans cell Histiocytosis (LCH) is a rare malignant disease. The clinical course is highly variable, ranging from self-limiting local disease to a rapidly progressive multisystem disorder that may lead to death [1]. A mutation in the BRAF gene, creating a BRAF^V600E^ mutant protein, can be found in a number of malignant diseases and is considered a driver mutation in a proportion of LCH patients [2, 3]. The mutation is associated with risk organ involvement, a more severe course of disease, poorer response to therapy, as well as a higher risk of disease relapse [4–6]. Although chemotherapy is the mainstay of LCH treatment, detection of BRAF mutation extends therapeutic options including selective BRAF inhibitors, such as vemurafenib [3]. The compound is not approved for this indication, but several reports have suggested its efficacy in patients with LCH [6–12]. Although vemurafenib seems to be a potent drug in order to stabilize the clinical condition of these patients, current data suggest that vemurafenib monotherapy cannot cure patients with LCH. In addition, to date, the optimal treatment duration with vemurafenib remains poorly defined, as well as whether adding chemotherapy to vemurafenib or replacing the compound with chemotherapy is of any benefit. Interestingly, measurement of circulating cell-free DNA of BRAF^V600E^ mutant alleles in peripheral blood has been reported as a promising biomarker in LCH, but it is unclear whether the assessment could help in decision making regarding vemurafenib therapy [6].

## CASE REPORT

**Figure 1:**
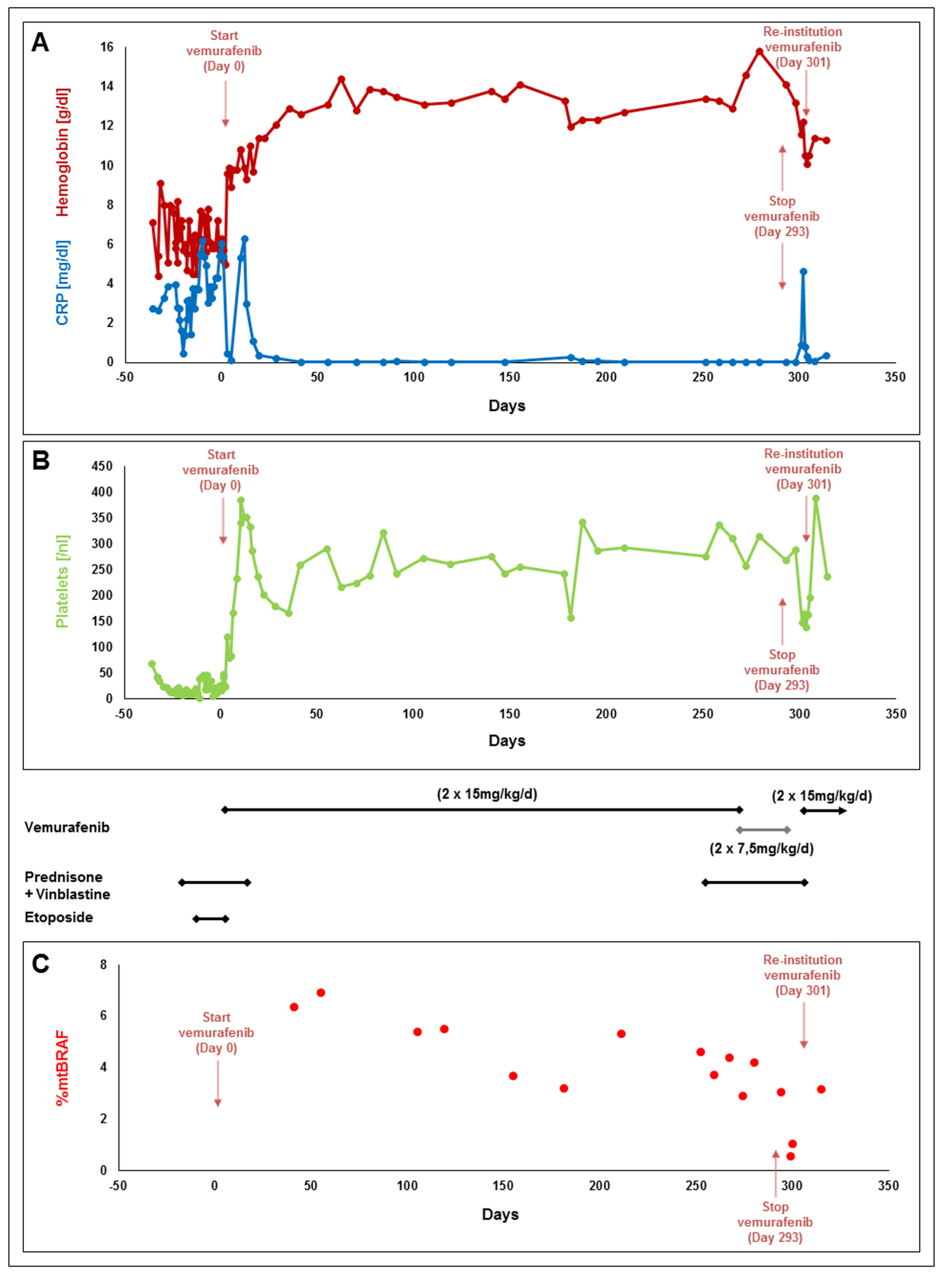
Levels of hemoglobin and C-reactive protein (CRP) **(A)**, platelets **(B)** and percentage of the BRAF V600E cells in the peripheral blood **(C)** of a patient with severe multisystem Langerhans cell Histiocytosis receiving different treatment regimens including vemurafenib.

A 2 3/12-year-old girl was admitted to the hospital in poor general condition with persisting fever of unknown origin. The previous history of the patient and the family was uneventful. Clinical examination revealed cervical lymphadenopathy, scaly retro-auricular skin lesions and hepatosplenomegaly (3 cm and 5 cm below costal margin, respectively). Laboratory findings demonstrated pancytopenia (hemoglobin 7.1 g/dl, leucocytes 3.23/nl, platelets 68/nl), elevated inflammation markers (C-reactive protein 2.74 mg/dl, soluble IL-2 receptor (sCD25) >22,500U/ml) and low total protein (5.3 g/dl). No malignant cells were detected in the bone marrow. Despite empirical therapy with broad-spectrum antibiotics, immunoglobulins and methyl-prednisolone, the clinical situation rapidly deteriorated [disease activity score (DAS) 19] (Figure 1A and 1B) [13]. LCH was diagnosed by histopathological and immunohistochemical examination of the cervical lymph node, but despite the administration of prednisone, vinblastine and etoposide, the clinical condition further aggravated and the patient required daily transfusions of red blood cells, platelets and albumin. After the BRAF^V600E^ mutation was demonstrated in the biopsy specimen, vemurafenib (15 mg/kg twice daily) was initiated, which resulted in a rapid clinical improvement. Within several days, the girl defervesced, liver and spleen almost normalized in size, and no further transfusions were required (DAS 2).

Over the next months, the girl stayed on vemurafenib monotherapy, which was well tolerated except for mild photosensitivity and alopecia. With informed consent of the parents, DNA was isolated from whole blood using the QIAamp DNA blood mini kit (Qiagen, Germany) and allele-specific PCR was performed at irregular time points to assess levels of BRAF mutant alleles which were slowly decreasing (Figure 1C) [12]. After 8 months of stable DAS of 1, we thought to stop vemurafenib due to the unknown long-term side effects. However, we aimed to replace vemurafenib by conventional LCH treatment with prednisone (40 mg/m^2^/d) and vinblastine (6 mg/m^2^/week). Therefore, we added both compounds while sustaining vemurafenib therapy, which was then tapered and finally stopped after 7 weeks of combination treatment. One week after cessation of vemurafenib, the girl developed fever and hepatosplenomegaly, and laboratory evaluation demonstrated pancytopenia and rising inflammatory markers. Vemurafenib treatment was re-initiated, resulting in a second complete remission and normal laboratory findings within several days. The girl is currently being prepared for allogeneic hematopoietic stem cell transplantation.

## DISCUSSION

The selective BRAF kinase inhibitor vemurafenib may be an effective therapeutic option in diseases with a BRAF^V600E^ mutation, which can be detected in almost 60% of patients with LCH [3]. To date, there are published reports on 7 adolescents older than 16 years and adults as well as on 7 children with LCH receiving vemurafenib (Table 1) [6–12]. All of them experienced a rapid partial or even complete clinical response. While these data seem encouraging, there may be a substantial publication bias favoring an efficacy of vemurafenib. Still, our patient responded exceedingly well. The patients reported received vemurafenib between 3 months up to 40 months, but it has to be noted that the optimal duration of therapy with vemurafenib remains unclear. Vemurafenib was discontinued in 7 patients, all of them developed disease reactivation. As the compound exerts skin and liver toxicity, is associated with increased risk of squamous cell carcinoma and as there are no data on the long-term risks, in particular in young children, we sought to terminate vemurafenib treatment [14]. In order to prevent disease reactivation, we first added standard chemotherapy with prednisone and vinblastine, and tapered vemurafenib thereafter, an approach which has not been reported to date. The combination of vemurafenib with LCH standard chemotherapy was well tolerated. No reactivation occurred during the tapering phase. However, after stopping vemurafenib, the patient relapsed within several days, indicating that standard chemotherapy was not useful to suppress or even to eradicate the LCH BRAF^V600E^ positive clone and thus to prevent relapse. In contrast, in this situation, re-institution of vemurafenib treatment was effective, as it was reported in all comparable patients (Table 1) [6, 11, 12]. Whether a more intensive chemotherapy, e.g. the administration of cytarabine or 2-CDA might prevent relapse with acceptable toxicity needs to be addressed in the future, but it might be possible that in these patients, conventional therapy will not have an effect at all.

**Table 1:** Published data on patients receiving vemurafenib for Langerhans cell Histiocytosis (LCH)

Author, year	Number of Patients; age/gender	Disease extent	Treatment prior to Vemurafenib (Duration)	Vemurafenib treatment				Current / last reported Status
				Dose and duration	Response to vemurafenib	Treatment duration/Discontinuation of vemurafenib	Relapse after discontinuation	
Bubolz et al. 2014	1 45 yrs/f	Multisystem	1) Steroids + VBL (1month, repeated after 4 months) 2) Cladribine (3 months)	240 mg, increased to 960mg	Partial	3 months / Yes	Yes	Progression of disease
Charles et al. 2014	1 >90 yrs/f	Skin	1) Steroids (NS) 2) Thalidomide (NS)	960mg	Complete	>6 months / No		Remission
Diamond et al. 2017	4 > 16 yrs/NS	NS	NS	All 960mg	Complete (1 pt) Partial (3 pts)	15-40 months / NS		1x CML 1x thyroid cancer 2x remission
Gandolfi et al. 2015	1 40 yrs/f	Skin, skull	1) Radiotherapy (30Gy) (NS) 2) MACOP-B (NS) 3) VNCOP-B (NS) 4) IEV (2 cycles) 5) BEAM (NS) 6) Bendamustine (6 cycles)	1920mg, reduced to 1440mg	Partial	10 months / No		Progression on vemurafenib after 10 months
Héritier et al. 2015	1 8 mths/f	Multisystem	1) Steroids + VBL (2 inductions) 2) Cladribine (1 course)	33.8mg/kg, reduced to 8.5mg/kg	Complete	4 months / Yes	Yes	Remission after re-institution of vemurafenib
Héritier et al. 2017	5 <3 yrs/NS	Multisystem	Steroids + VBL (1 induction)	NS	Partial / complete	NS / Yes (4 pts) No (1 pt)	Yes (all of 4 pts)	Remission after re-institution of vemurafenib
Kolenova et al. 2017	1 15 mths/m	Multisystem	1) Steroids + VBL (6 weeks) 2) Maintenance (2 weeks) 2) Cladribine (4 cycles)	2x10mg/kg, increased to 2x15mg/kg, then reduced to 10mg/kg	Complete	8 months / Yes	Yes	Remission after re-institution of vemurafenib, then HSCT
Own patient	1 2 yrs/f	Multisystem	Steroids + VBL (4 weeks) Etoposide	2x15 mg/kg	Complete	8 months / Yes	Yes	Remission after re-institution of vemurafenib

Yrs years; mths months; f female; m male; NS not specified; VBL vinblastine; pt(s) patient(s); MACOP-B doxorubicin, methotrexate, cyclophosphamide, vincristine, prednisone, bleomycin; VNCOP-B doxorubicin, cyclophosphamide, vincristine, prednisone, bleomycin; IEV ifosfamide, epirubicin, etoposide; BEAM carmustine, etoposide, cytarabine, melphalan; Maintenance mercaptopurine vinblastine, prednisolone; CML chronic myeloid leukemia; HSCT hematopoietic stem cell transplantation.

The level of BRAF mutant alleles in peripheral blood has been suggested as a marker for therapeutic response and may help to guide therapy [6, 12]. In our patient, BRAF mutant alleles remained detectable in peripheral blood throughout, even in the clinically stable patient after several months of vemurafenib treatment. BRAF mutant alleles were not quantified in the bone marrow, and it remains unresolved where the LCH clone was which caused relapse. It is unclear whether continuously positive BRAF levels in peripheral blood indicate highest risk of relapse. The fact that BRAF levels further decreased after vemurafenib was stopped and the patient already developed clinical signs of disease reactivation suggests that BRAF levels have a considerable time lag. On the other hand, BRAF levels were lowest after combination treatment with vemurafenib, prednisone and vinblastine, which might indicate that this regimen was more effective than vemurafenib alone, and one is prompted to speculate whether prolonged combination treatment could have prevented relapse. Nevertheless, it remains to be evaluated whether the assessment of BRAF levels at certain time points of therapy might help to stratify the intensity of treatment as it has been shown for minimal residual disease in acute lymphoblastic leukemia [15].

In conclusion, vemurafenib seems to be a powerful treatment option in patients with severe multisystem LCH, even during relapse. Further investigation needs to address 1) the optimal duration of vemurafenib therapy, 2) whether and which chemotherapeutic regimen may help to prevent disease relapse after cessation of vemurafenib and 3) the value of BRAF levels in guiding therapy.
